# Experimental Validation of Numerical Model for Thermomechanical Performance of Material Extrusion Additive Manufacturing Process: Effect of Process Parameters

**DOI:** 10.3390/polym14173482

**Published:** 2022-08-26

**Authors:** Ans Al Rashid, Muammer Koç

**Affiliations:** 1 Division of Sustainable Development, College of Science and Engineering, Hamad Bin Khalifa University, Qatar Foundation, Doha 34110, Qatar; 2 Faculty of Engineering, University of Karabük, Karabük 78050, Turkey

**Keywords:** material extrusion, additive manufacturing, fused filament fabrication, process simulation, residual stresses, warpage

## Abstract

The material extrusion additive manufacturing (MEAM) process for polymers seems straightforward. However, several controlled and uncontrolled factors affect the 3D printed product quality, e.g., MEAM process parameters, thermomechanical properties of the material, and part design. Therefore, it is crucial to understand these interlinked factors of part geometry, material properties, and 3D printing (3DP) process parameters to optimize 3D printed product quality. The numerical models and simulation tools can predict the thermomechanical performance of the MEAM process under given input parameters (material, design, and process variables) and reduce the research and development costs significantly. However, the numerical models and tools need further exploration and validation of simulation predictions for their adaptability and reliability. Therefore, in this study, numerical simulations were performed to observe the impact of process parameters on the part quality of MEAM 3D printed components. The two crucial process parameters (i.e., extrusion temperature and layer resolution) were varied while keeping the other process parameters, part geometry (tensile testing coupon), and material properties (acrylonitrile butadiene styrene (ABS)) constant. These two process parameters were sequentially optimized for optimum part quality, first by varying the extrusion temperature and secondly by changing the printing resolution using the optimum printing temperature. The 3DP process quality was evaluated in terms of dimensional accuracy, distortions, and residual stresses. Finally, the specimens were 3D printed under similar process conditions to validate the numerical model predictions.

## 1. Introduction

The material extrusion additive manufacturing (MEAM), also referred to as the fused filament fabrication (FFF) process, is one of the most widely explored and adopted additive manufacturing (AM) or 3D printing (3DP) processes owing to its simplicity, cheaper hardware, and open-source technology [1,2]. According to a study, in 2016, 96% of the global AM sales market was shared by the FFF-based 3D printers [3]. In this process, the 3D printer is fed with the material in filament form, heated to a semi-solid state, and extruded on a build plate through a nozzle [4]. The material is extruded layer-by-layer until a solid 3D part is fabricated, and the extrudate cools down on the build plate once the process is completed [5]. Thermoplastics are mainly processed using this process. However, the technology has recently evolved to use polymer composites [6] and metals [7].

Although the MEAM process for polymers seems straightforward, several controlled and uncontrolled factors affect the 3D printed product quality [8]. These factors include MEAM process parameters (e.g., nozzle diameter, deposition speed, layer resolution, raster pattern, raster width, infill type and density, material flow rate, raster orientation, building orientation, extrusion temperature, ambient temperature, and build plate temperature), thermomechanical properties of the material (e.g., thermal conductivity, heat capacity, young modulus, coefficient of thermal expansion, and stiffness), and part design [9,10,11]. Therefore, it is crucial to understand these interlinked factors of part geometry, material properties, and 3DP process parameters to optimize 3D printed product quality [12]. Several studies reported the experimental evaluation of the effect of these factors on the 3DP process quality [13,14,15]. Although several studies followed the experimental approach for this cause, the numerical modeling and simulations in this regard are very few.

The numerical models and simulation tools can predict the thermomechanical performance of the MEAM process under given input parameters (material, design, and process variables) and reduce the research and development costs significantly [16]. Computational fluid dynamics (CFD) models have been used to simulate the 3DP process and to analyze the temperature profile [17], material melting, layer adhesion, nozzle pressure, material flow, and heat transfer using different commercial software [18,19,20,21,22,23,24,25,26,27]. However, the numerical models and tools need further exploration and validation of simulation predictions for their adaptability and reliability.

In the view of existing literature, it is vital to understand and evaluate the effect of MEAM process parameters on the 3D printed product quality. The reported study is part of our ongoing research where all aspects of the 3DP process, i.e., material properties, process parameters, and the design of specimens, are being considered. Other aspects will also be covered in our upcoming studies. However, the effect of extrusion temperature and layer height is investigated in this study. Therefore, numerical simulations were performed to observe the impact of process parameters on the part quality of MEAM 3D printed components. Different extrusion temperatures (210 °C, 220 °C, 230 °C, 240 °C, 250 °C, and 260 °C) within the printability window of acrylonitrile butadiene styrene (ABS) material and three different layer heights (0.1 mm, 0.15 mm, and 0.2 mm) were selected for analysis. These two process parameters were sequentially optimized for optimum part quality, first by varying the extrusion temperature and secondly by changing the printing resolution using the optimum printing temperature. The 3DP process quality was evaluated regarding dimensional accuracy, distortions, and residual stresses. Finally, the specimens were 3D printed under similar process conditions to validate the numerical model predictions.

## 2. Methodology

**Figure 1:**
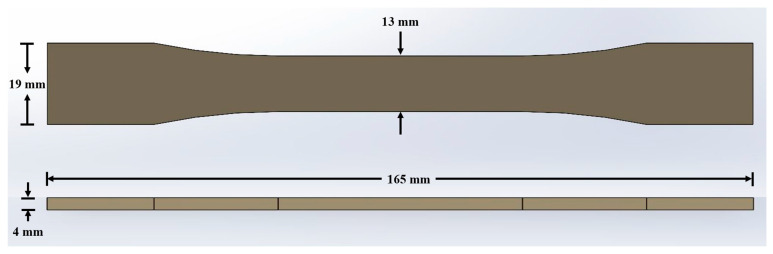
CAD Model for ASTM D638 type I specimen with dimensions.

The current work is divided into two phases: the MEAM process numerical simulations and validation of numerical model results via 3DP experimental measurements. The main aim of this study is to evaluate the effect of process parameters on 3DP process quality quantified in terms of part deflections, distortions, and residual stresses. The extrusion temperature and printing resolution were varied while keeping the other process parameters, part geometry, and material properties constant. A standardized tensile testing coupon (according to ASTM D638 Type-I [28]) was selected as reference geometry to be fabricated using acrylonitrile butadiene styrene (ABS) material (from Ultimaker, The Netherlands). ABS provides flexibility in synthesizing nanocomposites due to its solubility in acetone [29] and possesses significant physical, mechanical, and chemical resistance properties [30,31]. A 3D CAD model for a tensile testing coupon was generated using Solidworks 2021 (Dassault Systèmes, Paris, France), as shown in Figure 1, and imported to slicing software. The slicing information (gcode) was generated using Cura software version 5.0.0 (from Ultimaker, The Netherlands), containing information on toolpath, printing resolution, extrusion temperature, build plate temperature, printing speed, and infill type and density. The subsequent sections present the methods adopted for numerical simulations and experimentation.

### 2.1. MEAM Process Simulations

The MEAM process simulations were performed using Digimat software (version 2021.3, from e-Xstream engineering, Käerjeng, Luxembourg). Chamber size of 215 mm (X) × 215 mm (Y) × 200 mm (Z) was defined with a moving build plate corresponding to the Ultimaker 3 Extended 3D printer (from Ultimaker, The Netherlands). The software utilizes a thermomechanical numerical model to calculate the temperature variations during the MEAM process [32]. Heat transfer equations evaluate the time-dependent temperature fields layer-by-layer during the material deposition. The built plate and ambient temperatures are used as boundary conditions for bottom surface elements and ambient environment for the overall model, respectively. The first deposited layer is constrained in all degrees of freedom due to the build plate adhesion, which gets released at the end of the 3DP process, and final residual stresses and deformations are estimated in this state.

The thermal gradients due to convective and radiative heat transfer cause the material shrinkage, which results in part deformations and distortions [33]. However, the residual stresses arise due to restricted molecular movements due to structural constraints [34]. The 3D model of the tensile testing coupon was imported in stl format, and thermomechanical properties (for ABS material) were defined from the software database. The numerical model requires toolpath information for 3D printed specimens imported as a gcode file. Process parameters were specified, including extrusion temperature (210 °C, 220 °C, 230 °C, 240 °C, 250 °C, and 260 °C), build plate temperature (80 °C), bead width (0.4 mm), chamber temperature (25 °C), convection coefficient (0.015 mW/mm^2^·°C), and ambient temperature (25 °C). Except for extrusion temperature and printing resolution, the other process parameters were constant for all the simulations. Voxel size (0.1, 0.15, or 0.2) was selected per the layer heights. Digimat software provides two options for geometry discretization, i.e., filament or layer-by-layer. Although filament discretization offers more precise results, the layer-by-layer technique was used to lower the computational time and cost.

### 2.2. MEAM Experiments

Ultimaker 3 Extended 3D printer and ABS material in filament form (from Ultimaker, The Netherlands) were used for 3DP experiments. The slicing information (gcode) was generated using Cura software (from Ultimaker, The Netherlands), containing information on toolpath, printing resolution, extrusion temperature, build plate temperature, printing speed, and infill type and density. Only two process parameters, i.e., extrusion temperature and printing resolution, were varied for their sensitivity to product quality. Different extrusion temperatures (210 °C, 220 °C, 230 °C, 240 °C, 250 °C, and 260 °C) within the printability window of ABS material and three different layer heights (0.1 mm, 0.15 mm, and 0.2 mm) were selected to fabricate 3D printed samples. These two process parameters were sequentially optimized for optimum part quality, first by varying the extrusion temperature and secondly by changing the printing resolution using the optimum printing temperature. The specimens were manufactured at a speed of 60 mm/s, using the build plate temperature of 80 °C, with two wall layers (corresponding to 0.8 mm of thickness as bead width was 0.4 mm). The top and bottom layers were 1.2 mm thick (where the number of layers varied depending upon the layer height), having 100% infill density with a zig-zag infill pattern.

The physical measurements were performed on the 3D printed samples for their dimensions and distortions. The dimensions were recorded using a vernier caliper, and at least three measurements were recorded for each targeted dimension. The measurements were compared with the numerical model predictions and designed part dimensions. Finally, the variations in residual stresses due to changes in 3DP process parameters were observed, and their effect on part quality was analyzed.

## 3. Results and Discussions

The numerical model performs the MEAM process simulation layer-by-layer to mimic the actual process, where a set of elements in the printing direction are activated in a time step. Depending upon the discretization approach, a group of elements is activated at a temperature selected for the extrusion process (ranging from 210 °C to 260 °C in this case). Due to convective and radiative heat losses, the extrudate temperature is significantly higher than the already deposited material [22]. As the printing process progresses, the deposited material cools down close to the printing platform temperature; though, the upcoming extrudate re-heats the already deposited material [35]. Due to the continuous thermal cycling of the material and heat transfer phenomena in the ambient environment, the thermal gradients are observed in the build direction, resulting in thermal stresses within the structure [33]. The temperature variations and material properties govern the thermal strains within the 3D printed part due to induced thermal stresses. The thermal strains result in dimensional variations of the 3D printed part from the designed dimensions [36]. Hence, the impact of 3DP process parameters, design, and material properties need to be investigated and understood to obtain desired structural integrity. The process parameters are sequentially optimized for ABS material to achieve optimum 3DP product quality by adjusting the extrusion temperature and printing resolution. The numerical model predictions were validated with 3DP experiments performed under the same process conditions.

### 3.1. Effect of Extrusion Temperature

**Figure 2:**
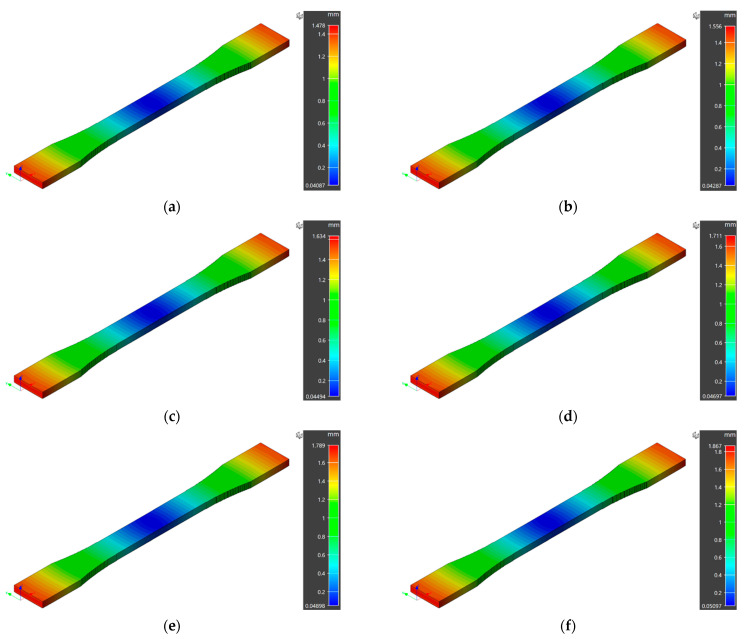
Maximum displacements in numerical model simulations at different extrusion temperatures: (**a**) 210 °C, (**b**) 220 °C, (**c**) 230 °C, (**d**) 240 °C, (**e**) 250 °C, (**f**) 260 °C.

The numerical model estimates the deviation of 3D printed sample dimensions from the designed values in three principal directions. The overall maximum displacements for the 3D printed samples are reported in Figure 2. The extrusion temperature varied from 210 °C to 260 °C within the processability window of the ABS material. The overall maximum displacement of 1.478 mm, 1.556 mm, 1.634 mm, 1.711 mm, 1.789 mm, and 1.867 mm was observed for specimens 3D printed at 210 °C, 220 °C, 230 °C, 240 °C, 250 °C, and 260 °C, respectively. In numerical predictions, a maximum deflection from designed dimensions was observed for specimen 3D printed at 260 °C. However, a minimum deviation was achieved for extrusion temperature of 210 °C. The coefficient of thermal expansion plays a vital role in material shrinkage, and the numerical simulation results also showed a prominent effect of printing temperature. In addition, the deformation trend for all the printing temperatures was the same, i.e., maximum at the specimen edges and minimum at the central region, owing to non-uniform cooling of the specimen edges.

#### 3.1.1. Dimensional Analysis

**Figure 3:**
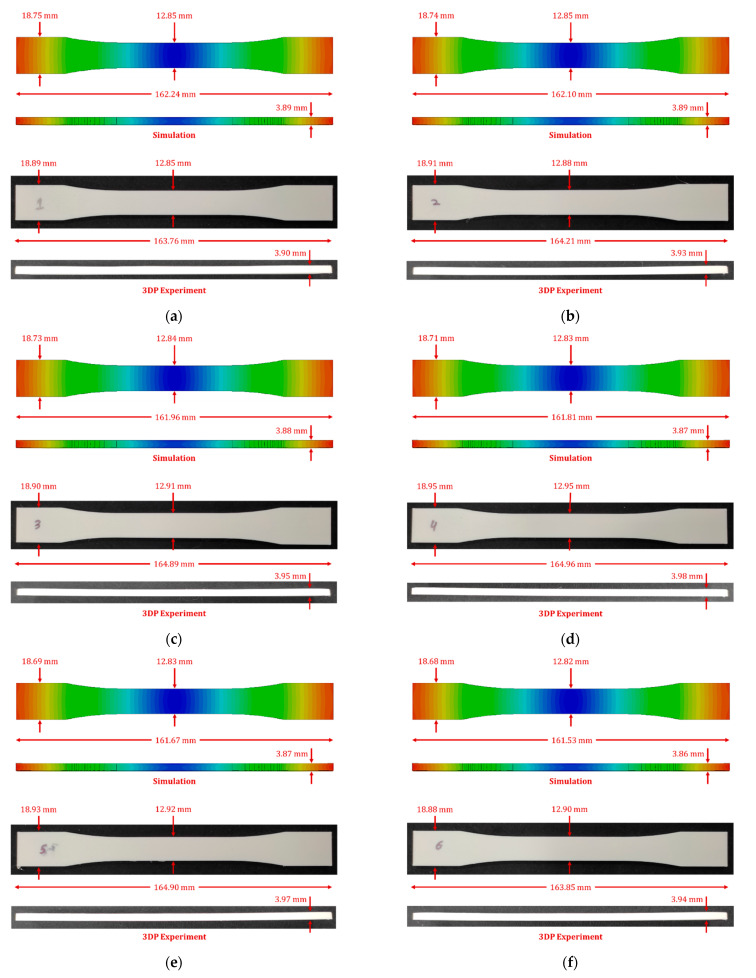
Specimen dimensions for numerically predicted and fabricated samples 3D printed at different temperatures: (**a**) 210 °C, (**b**) 220 °C, (**c**) 230 °C, (**d**) 240 °C, (**e**) 250 °C, (**f**) 260 °C.

The numerical simulation tool does not directly provide the dimensions of the simulated parts. Therefore, warped geometries were exported for dimensional analysis using Solidworks software 2021 (Dassault Systèmes, Paris, France). The targeted dimensions were measured as estimated by the numerical model and compared with the physical measurements taken for the 3D printed samples, as reported in Figure 3.

Table 1 comprehensively compares measurements recorded for the samples fabricated at different extrusion temperatures. Generally, the material shrinkage was observed in all the numerical simulation results in three principal directions. In numerical simulation results, a maximum deviation in overall length from the designed value of the specimen was 3.47 mm for 260 °C extrusion temperature. However, a minimum variation of 2.76 mm was achieved for specimen 3D printed at 210 °C. A maximum deviation of 0.32 mm in grip width from the designed value of the specimen was observed for 260 °C extrusion temperature. However, a minimum deviation of 0.25 mm was achieved for specimen 3D printed at 210 °C. Similarly, 0.15 mm and 0.18 mm of variation from the designed value of gauge width was measured for extrusion temperatures of 210 °C and 260 °C. Finally, the specimen thickness of 3.89 mm was achieved at printing temperatures of 210 °C and 220 °C, although a thickness of 3.86 mm was recorded for samples 3D printed at 260 °C. However, the numerical model predicted higher deflections from the designed values than the experimental measurements. In addition, a different trend in the dimensional measurements was observed, where the overall 3D printed specimen at 240 °C revealed better dimensional control over the rest of the extrusion temperatures considered.

In experimental measurements, a maximum deviation in overall length from the designed value of the specimen was 1.24 mm for 210 °C extrusion temperature. However, a minimum variation of 0.04 mm was achieved for specimen 3D printed at 240 °C. A maximum deviation of 0.12 mm in grip width from the designed value of the specimen was observed for 260 °C extrusion temperature. However, a minimum deviation of 0.05 mm was achieved for specimen 3D printed at 240 °C. Similarly, 0.05 mm and 0.15 mm of variation from the designed value of gauge width was measured for extrusion temperatures of 240 °C and 210 °C. Finally, the specimen thickness of 3.98 mm was achieved at printing temperatures of 240 °C. However, a thickness of 3.90 mm was recorded for samples 3D printed at 210 °C.

Overall, the numerical model predicted the measurements near the experimental dimensions. However, the higher deflections from the designed values with increasing extrusion temperatures are attributed to the material model limitation in the existing model. The material model currently does not consider the temperature dependence of the material properties, resulting in a linear trend with decaying product quality due to increased extrusion temperature. Secondly, higher variations in numerical model predictions are observed due to higher material shrinkages. This can be attributed to the stress relaxation phenomena in the 3D printed samples, which lowers the residual stresses and deflections; nevertheless, the stress relaxation is not considered in the current numerical model; hence higher shrinkages are observed. Secondly, the printing temperature also played an essential role in dimensional control of the 3D printed specimens, as higher variations are observed at higher extrusion temperatures. The experimental analysis concluded that optimum control over dimensions is achieved at an extrusion temperature ranging from 240 °C to 250 °C.

#### 3.1.2. Warpage Analysis

**Figure 4:**
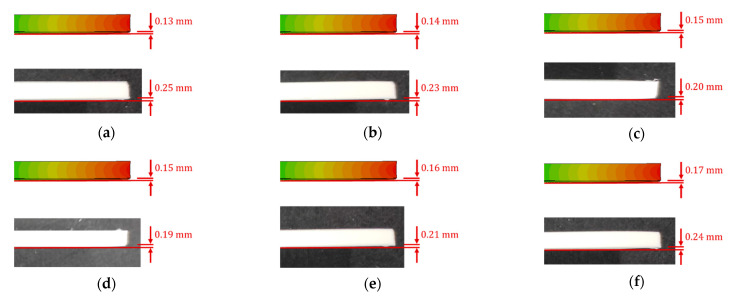
Numerically predicted (above) and physically measured (below) warpage for 3D printed samples at different temperatures: (**a**) 210 °C, (**b**) 220 °C, (**c**) 230 °C, (**d**) 240 °C, (**e**) 250 °C, (**f**) 260 °C.

Besides dimensional analysis, the out-of-plane deformations of the 3D printed parts are essential to analyze and are termed as distortions or warpage. The warpages come into action due to the thermal gradient in the build direction. In addition, the specimen edges go through non-uniform cooling, resulting in higher thermal stresses in these regions. The warpage measurements recorded from the numerical model predictions and 3D printed samples are also reported in Table 1. A visual comparison of warpages for simulation and experimental results is reported in Figure 4. The numerical model results recorded maximum and minimum warpage of 0.17 mm and 0.13 mm for specimens fabricated at 260 °C and 210 °C, respectively.

Although the differences in warpage measurements are a fraction of millimeters, they are important to consider where tighter control over dimensions is desired. The measured warpage in the 3D printed samples revealed higher values than the numerically predicted results. The imperfect build plate adhesion resulted in this variation, as the numerical model assumes perfect adhesion to the build plate. However, thermal stresses developed due to non-uniform cooling of the specimen edges, resulting in part detachment from the build plate. Therefore, higher z-direction deflections are observed. The numerical model predictions show that minimum warpage is achieved at an extrusion temperature of 210 °C. However, in experimental measurements, minimum warpage is achieved at 240 °C. The numerical model revealed a similar trend to deflections, where the warpages increase with increased extrusion temperatures. This is once again attributed to the existing material model limitations. Contrary to experimental measurements, higher warpages at higher or lower temperatures than the optimum temperature are due to two main reasons. Once the material is deposited at a temperature lower than the optimum temperature of 240 °C, it tends to cool down to the build plate or ambient temperature, depending on the layer number [37,38]. As the material cools down, the phase-change process from semi-solid to solid starts due to lower extrusion temperature, and inter-bead diffusivity of polymer chains is inhibited, resulting in imperfect adhesion. However, higher thermal cycling increases residual strains and warpages at higher temperatures.

#### 3.1.3. Residual Stresses

**Figure 5:**
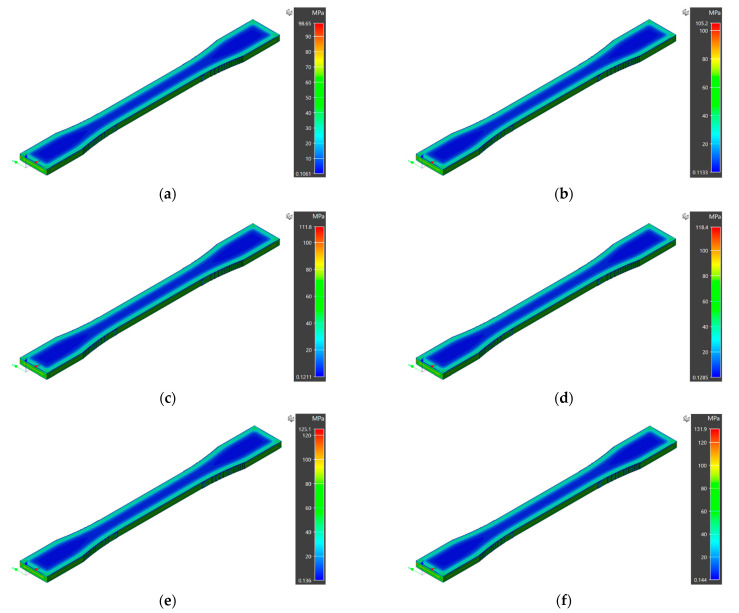
Residual stresses in numerical model simulations at different extrusion temperatures: (**a**) 210 °C, (**b**) 220 °C, (**c**) 230 °C, (**d**) 240 °C, (**e**) 250 °C, (**f**) 260 °C.

The numerical model also predicted the residual stresses generated within the 3D printed part due to thermal cycling and process constraints. The numerically predicted residual stresses for the specimens manufactured at different extrusion temperatures are presented in Figure 5. The thermal gradient along the z-direction and non-uniform cooling of the samples results in the residual stresses.

Generally, higher residual stresses are generated along the specimen edges, and significantly lower residual stresses are observed within the specimen structure. In addition, the specimen 3D printed at an extrusion temperature of 210 °C revealed lower residual stresses than all the other printing temperatures under consideration. This observation also supports numerical predictions of the higher dimensional control at an extrusion temperature of 210 °C, as higher residual stresses result in higher dimensional variations and distortions in the specimen. The residual stresses can be limited by controlling the cooling process. Hence, the 3DP process within the temperature-controlled closed chamber can provide better quality.

### 3.2. Effect of Layer Height

In the second phase, only numerical simulations were performed to evaluate the effect of layer height on 3DP quality. Three different layer heights (0.10 mm, 0.15 mm, and 0.20 mm) were selected with an optimum extrusion temperature of 240 °C, while all the other process parameters were kept constant. The voxel sizes varied accordingly to mimic the layering effect and height. The process simulation for 0.20 mm layer height was performed in the previous phase. Only two simulations for 0.10 mm and 0.15 mm of layer resolution were performed, and results are reported in the subsequent sections.

**Figure 6:**
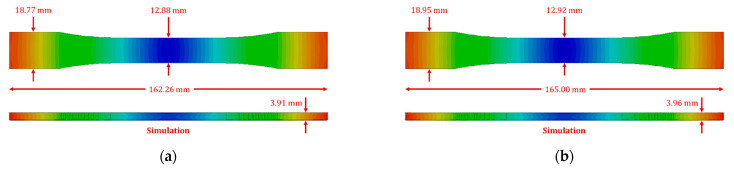
Specimen dimensions for numerically predicted 3D printed at different layer heights: (**a**) 0.15 mm, (**b**) 0.10 mm.

**Figure 7:**
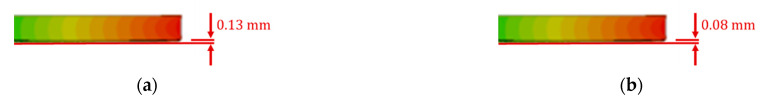
Numerically predicted warpage for 3D printed samples at different layer heights: (**a**) 0.15 mm, (**b**) 0.10 mm.

The dimensional and distortion analysis was performed on the numerically predicted results, as reported in Table 2. A significant improvement in dimensional control was achieved by reducing the layer height. A comparison of dimensions for specimens virtually fabricated at layer heights of 0.15 mm and 0.10 mm is presented in Figure 6. An overall length of 162.26 mm and 165.00 mm, grip width of 18.77 mm and 18.95 mm, the gauge width of 12.88 mm and 12.92 mm, and thickness of 3.91 mm and 3.96 mm was achieved for 0.15 mm and 0.10 mm of layer heights, respectively. Finally, a warpage of 0.13 mm and 0.08 mm was observed for specimens modeled at 0.15 mm and 0.10 mm of printing resolution, as reported in Figure 7.

The results show that, at higher printing resolutions (lower layer heights), higher dimensional accuracy of the product is achieved. The 0.10 mm layer height produced specimens with final dimensions close to desired ones. As the layer height reduces (increasing resolution), the number of layers producing a 4 mm thickness increases. Therefore, the cooling of a layer with 0.10 mm has enough time to adequately bond to the already deposited layer and solidifies at the deposited position before the deposition of the incoming layer. Consequently, improved dimensional accuracy is achieved.

## 4. Conclusions

In this study, numerical simulations were performed to observe the impact of process parameters on the part quality of MEAM 3D printed components. Different extrusion temperatures (210 °C, 220 °C, 230 °C, 240 °C, 250 °C, and 260 °C) within the printability window of ABS material and three different layer heights (0.1 mm, 0.15 mm, and 0.2 mm) were selected for analysis. These two process parameters were sequentially optimized for optimum part quality, first by varying the extrusion temperature and secondly by changing the printing resolution using the optimum printing temperature. The 3DP process quality was evaluated regarding dimensional accuracy, distortions, and residual stresses. Finally, the specimens were 3D printed under similar process conditions to validate the numerical model predictions.

The numerical model predicted the actual 3DP process outcomes with a reasonable difference from the experimental results. However, the higher deflections from the designed values with increasing extrusion temperatures are attributed to the material model limitation in the existing model. The higher dimensional variations in numerical model results can also be attributed to the stress relaxation effect, which is not considered in the numerical model. However, higher warpages/distortions recorded in 3D printed samples were due to the numerical model considering perfect build plate adhesion of the specimen. The 3DP experimental results conclude that the optimum print quality can be achieved at an optimum extrusion temperature of 240 °C. Two main reasons are that higher-dimensional variations and warpages at higher or lower temperatures than the optimum temperature. Once the material is deposited at a temperature lower than the optimum temperature of 240 °C, it tends to cool down to the build plate or ambient temperature, depending on the layer number. As the material cools down, the phase-change process from semi-solid to solid starts due to lower extrusion temperature, and inter-bead diffusivity of polymer chains is inhibited, resulting in imperfect adhesion. However, higher thermal cycling increases residual strains and warpages at higher temperatures. The higher thermal gradients within the printed part can also lead to higher distortions in the build direction.

In addition, the specimen 3D printed at an extrusion temperature of 210 °C revealed lower residual stresses than all the other printing temperatures under consideration. This observation also supports numerical predictions of the higher dimensional control at an extrusion temperature of 210 °C, as higher residual stresses result in higher dimensional variations and distortions in the specimen. Finally, the printing resolution also plays a vital role in the dimensional control of 3D printed parts. Based on the numerical model predictions, it is suggested that higher printing resolutions should be used to produce higher quality parts, especially in functional components where higher dimensional control is desired.

It is concluded that extrusion temperature and process resolution are of paramount concern while achieving optimum product quality. The optimum printing temperature varies with material compositions. However, lower layer heights are suggested for higher dimensional accuracy of 3D printed parts. The presented numerical model can be further improved to optimize the printing temperature and layer heights for other materials. In future studies, other polymeric materials will be considered to optimize the process parameters for the MEAM process. The proposed methodology can be implemented at a lower cost and limit the need for experimental optimization of 3DP process parameters.

## Figures and Tables

**Table 1:** Effect of extrusion temperature on dimensional variations in numerical model and 3DP parts.

Run	Extrusion Temperature (°C)	Model	Length (mm)	Grip Width (mm)	Gauge Width (mm)	Thickness (mm)	Warpage
-	-	Design	165.00	19.00	13.00	4.00	-
1	210	Numerical	162.24 ± 0.01	18.75 ± 0.02	12.85 ± 0.02	3.89 ± 0.02	0.13 ± 0.01
		3DP	163.76 ± 0.01	18.89 ± 0.01	12.85 ± 0.01	3.90 ± 0.02	0.25 ± 0.01
2	220	Numerical	162.10 ± 0.01	18.74 ± 0.02	12.85 ± 0.01	3.89 ± 0.02	0.14 ± 0.01
		3DP	164.21 ± 0.01	18.91 ± 0.02	12.88 ± 0.02	3.93 ± 0.02	0.23 ± 0.01
3	230	Numerical	161.96 ± 0.01	18.73 ± 0.02	12.84 ± 0.01	3.88 ± 0.02	0.15 ± 0.01
		3DP	164.89 ± 0.01	18.90 ± 0.02	12.91 ± 0.02	3.95 ± 0.02	0.20 ± 0.01
4	240	Numerical	161.81 ± 0.02	18.71 ± 0.02	12.83 ± 0.02	3.87 ± 0.02	0.15 ± 0.01
		3DP	164.96 ± 0.01	18.95 ± 0.02	12.95 ± 0.02	3.98 ± 0.02	0.19 ± 0.01
5	250	Numerical	161.67 ± 0.02	18.69 ± 0.02	12.83 ± 0.01	3.87 ± 0.02	0.16 ± 0.01
		3DP	164.90 ± 0.01	18.93 ± 0.02	12.92 ± 0.02	3.97 ± 0.02	0.21 ± 0.01
6	260	Numerical	161.53 ± 0.02	18.68 ± 0.02	12.82 ± 0.01	3.86 ± 0.02	0.17 ± 0.01
		3DP	163.85 ± 0.01	18.88 ± 0.02	12.90 ± 0.01	3.94 ± 0.02	0.24 ± 0.01

**Table 2:** Effect of Layer Height on Dimensional Variations in Numerical Model and 3DP Parts.

Run	Layer Height (mm)	Model	Length (mm)	Grip Width (mm)	Gauge Width (mm)	Thickness (mm)	Warpage
-	-	Design	165.00	19.00	13.00	4.00	-
1	0.20	Numerical	161.81 ± 0.02	18.71 ± 0.02	12.83 ± 0.02	3.87 ± 0.02	0.15 ± 0.01
2	0.15	Numerical	162.26 ± 0.01	18.77 ± 0.01	12.88 ± 0.01	3.91 ± 0.02	0.13 ± 0.01
3	0.10	Numerical	165.00 ± 0.01	18.95 ± 0.01	12.92 ± 0.01	3.96 ± 0.01	0.08 ± 0.01

## Data Availability

The data presented in this study are available on request from the corresponding author.
